# The Janus Face of VEGF in Stroke

**DOI:** 10.3390/ijms19051362

**Published:** 2018-05-04

**Authors:** Samuel J. Geiseler, Cecilie Morland

**Affiliations:** 1 Department of Pharmaceutical Biosciences, School of Pharmacy, University of Oslo, 0371 Oslo, Norway; samuel.geiseler@farmasi.uio.no; 2 Institute for Behavioral Sciences, Faculty of Health Sciences, OsloMet—Oslo Metropolitan University, 0166 Oslo, Norway

**Keywords:** growth factor, VEGF, stroke, ischemia, exercise

## Abstract

The family of vascular endothelial growth factors (VEGFs) are known for their regulation of vascularization. In the brain, VEGFs are important regulators of angiogenesis, neuroprotection and neurogenesis. Dysregulation of VEGFs is involved in a large number of neurodegenerative diseases and acute neurological insults, including stroke. Stroke is the main cause of acquired disabilities, and normally results from an occlusion of a cerebral artery or a hemorrhage, both leading to focal ischemia. Neurons in the ischemic core rapidly undergo necrosis. Cells in the penumbra are exposed to ischemia, but may be rescued if adequate perfusion is restored in time. The neuroprotective and angiogenic effects of VEGFs would theoretically make VEGFs ideal candidates for drug therapy in stroke. However, contradictory to what one might expect, endogenously upregulated levels of VEGF as well as the administration of exogenous VEGF is detrimental in acute stroke. This is probably due to VEGF-mediated blood–brain-barrier breakdown and vascular leakage, leading to edema and increased intracranial pressure as well as neuroinflammation. The key to understanding this Janus face of VEGF function in stroke may lie in the timing; the harmful effect of VEGFs on vessel integrity is transient, as both VEGF preconditioning and increased VEGF after the acute phase has a neuroprotective effect. The present review discusses the multifaceted action of VEGFs in stroke prevention and therapy.

## 1. Introduction

Millions of people suffer a stroke every year, and stroke is the main cause of disabilities among adults. The two major causes of stroke are an occlusion of a precerebral or cerebral artery or a hemorrhage. Both cause ischemia, which rapidly leads to necrosis in the stroke core. The fate of the area surrounding the necrotic core, the penumbra, depends largely on the time before reperfusion is restored. Neural death is proportional to the degree of loss in perfusion, and early reperfusion is essential to prevent extensive neural damage [1,2,3,4]. Brain injury after stroke occurs as a result of a complex series of pathophysiological events, including excitotoxicity, oxidative stress, vasopermeability of the blood–brain barrier (BBB), and inflammation, leading to cell death. Growth factors are important regulators of protection and recovery after ischemia, and the combined action of growth factors regulates angiogenesis, neuroprotection, neurogenesis as well as the migration of neuronal stem cells into the ischemic area, and their proliferation into functional neurons. One important family of growth factors is the family of vascular endothelial growth factors (VEGFs). Due to their upregulation in the ischemic brain and their strong angiogenic and neuroprotective properties [5,6,7,8,9], the administration of VEGFs per se, or substances that regulate VEGFs or VEGF receptor actions, are considered interesting potential treatment strategies in stroke.

The mammalian VEGF family consists of five members, VEGF-A, -B, -C, -D, and placental growth factor (PIGF). Of these, VEGF-A has gained most attention. VEGF-A is pro-angiogenic and neuroprotective and induces neurogenesis [10]. Therefore, systemic treatment with VEGF-A in stroke has gained considerable interest. However, such VEGF-A treatment has shown somewhat disappointing and inconsistent effects in pre-clinical trials. This is probably because VEGF-A has a dual role in the regulation of the vascular endothelium [11]: firstly, it stimulates the proliferation and migration of endothelial cells, leading to the formation of new vessels. This results in a more efficient network of collaterals, which may bypass the occluded vessel and thereby rescue the penumbra. However, VEGF-A also increases vascular permeability [12]. The latter effect may cause brain edema and increase intracranial pressure, which is detrimental in the acute phase of stroke [13]. Increased vascular permeability also allows the entry of molecules and immune cells that are normally prohibited by the blood–brain barrier (BBB), causing neuroinflammation.

## 2. The VEGF Receptor Family

The canonical VEGF signal is initiated when VEGFs bind to VEGF receptors, namely VEGFR-1 (fms-like tyrosine kinase-1; flt-1), VEGFR-2 (fetal liver kinase-1 (flk-1)/kinase domain receptor (KDR)) or VEGFR-3 (flt-4). These receptors belong to the family of receptor tyrosine kinases. The overall structure of these receptors, and the intracellular signaling cascades they initiate, are well preserved through evolution [14], indicating that they regulate important functions in development as well as in adult health and disease. Non-canonical VEGF signaling also exists, for example when VEGFs activate other receptors than the VEGFRs. One such receptor family is the family of neuropilin (NRP) receptors, which consists of NRP-1 and NRP-2. The NRPs are not “true” receptors, as they lack intrinsic enzymatic activity and therefore cannot be activated by the binding of agonists. These are termed co-receptors for the VEGFs. Members of the VEGF family bind to the VEGFRs or co-receptors with different affinities: VEGF-A primarily binds to VEGFR-1, VEGFR-2, NRP-1 and NRP-2, while VEGF-B binds to VEGFR-1 and NRP-1. VEGF-C and VEGF-D predominantly binds to VEGFR-3, VEGFR-2 and PIGF binds VEGFR-1, NRP-2 and NRP-1 (for review, see [15]).

The VEGF receptors and co-receptors have different properties and activate different intracellular signaling pathways. VEGF receptor signaling has been reviewed elsewhere in detail [16]. Although VEGF-A also activates other receptors, the main focus of this review is on the effects that are initiated by the binding of VEGF-A to VEGFR-2, as they are the most important in stroke. VEGFR-1 may act as a decoy receptor [17,18], the main effect of which is to prevent the binding of VEGF-A to VEGFR-2. Angiogenesis therefore requires a balanced action of VEGFR-1 and VEGFR-2. Depending on the residue of the intracellular phosphorylation, the activation of VEGFR-2 triggers different intracellular signaling cascades. Although these pathways regulate partly overlapping functions in the cell, each pathway can explain at least one of the main functions of VEGF in stroke. The phosphatidylinositol-3-kinases-protein/kinase B (PI3K/Akt) pathway is a main survival pathway, the MEK–MAPK pathway regulates proliferation and migration, and the Src-suppressed C kinase substrate (SSeCKS) pathway mediates vasopermeability, all of which are important effects of VEGF-A in stroke.

## 3. VEGF-A in the Prevention and Therapy of Stroke

### 3.1. VEGF-A Expression

In the healthy adult mammalian brain, VEGF-A is expressed at low levels and is observed sporadically in parenchymal cells in most brain regions [19]. VEGFR-2 is expressed on neurons and vascular endothelial cells [20,21,22], while VEGFR-1 is found in the vasculature, in the choroid plexus and on glial cells.

In response to stroke, VEGF-A as well as its receptors VEGFR-1 and VEGFR-2 are up-regulated [23,24]. The increase occurs predominantly in the stroke/penumbra area. However, an increase in the cortical areas that are functionally and/or behaviorally related to the area of the infarction has also been reported [25,26]. In stroke, VEGF-A is increased in astrocytes, neurons and endothelial cells, and in the penumbra, compared to both the infarcted area and the contralateral hemisphere [22,24,27,28,29]. As for VEGF-A itself, the expression of VEGFR-2 in the vasculature increases in the penumbra compared to the contralateral hemisphere [24]. VEGFR-1 increase has been reported in the penumbra, but also in the pia mater, and on the vessel invading the core region of the infarcted area [23]. In the penumbra, the upregulation of VEGFR-1 was almost exclusively in reactive astrocytes [30,31] and in sprouting vessels [23]. The increase in VEGF-A and VEGF-receptors is reported to start as early as 2–4 h after the onset of stroke and to last for at least 28 days [28]. The increase starts later in astrocytes than in neurons [32]. According to Zan and coworkers [33], the increase in VEGF-A in response to ischemia is biphasic. The authors reported a first peak in VEGF-A at 6 h after reperfusion that normalized within 12 h, only to peak again at seven days after reperfusion. VEGF-A levels were reported to return to baseline after two weeks.

What regulates VEGF-A levels in the brain in response to stroke? Hypoxia-inducible factor 1 (HIF-1) is a pivotal transcription factor in the hypoxic brain. It consists of an inducible subunit HIF-1α and a constitutive subunit, HIF-1β. Under normoxic conditions, HIF-1α is rapidly ubiqutinylated and degraded. However, hypoxia stabilizes HIF-1α, leading to an upregulation of HIF-1α in the hypoxic/ischemic brain [34,35]. HIF-1α is a prominent activator of VEGF-A gene expression [23,36]. The direct connection between HIF-1α and its target-gene VEGF-A in hypoxia/ischemia is well documented [37,38,39]. An indirect mechanism may also be involved, where HIF-1α induces erythropoietin (EPO) [40], which in turn increases the secretion of VEGF [41]. Inflammatory cytokines may also regulate VEGF-A in stroke [42,43]. In addition, we had recently shown that lactate increases VEGF-A expression via the lactate receptor HCAR1, which causes HCAR1-dependent angiogenesis in the brain [44].

### 3.2. VEGF-A in the Treatment of Stroke

The current state of knowledge regarding the role of VEGF-A in stroke is almost exclusively based on animal models. VEGF-A has multiple protective effects, including the promotion of angiogenesis, neurogenesis, and neuroprotection, leading to improved functional recovery [45]. VEGF-A is significantly upregulated in the brain of the naked mole rat (*Heterocephalus glaber*), where it contributes to their exceptional intrinsic hypoxia tolerance [46,47]. VEGF-A is therefore a very interesting candidate for therapeutic treatment in ischemic stroke [48]. The involvement of VEGF-A in protective and harmful mechanisms in stroke is discussed below.

#### 3.2.1. Effects of VEGF-A in Angiogenesis

Increased angiogenesis is highly important for the neuroprotective effects of VEGFs in stroke, and the upregulation of VEGF-A and VEGFR-2 in the penumbra is directly correlated to neuro-vascularization [27,28,49]. In the normal brain, the administration of VEGF-A causes an upregulation of VEGFR-1 and VEGFR-2 and a significant increase in cerebral vascularization [22]. Furthermore, the transplantation of stem cells that overexpress VEGF-A has been shown to cause angiogenesis of the host nervous tissue [50]. VEGF-A regulates angiogenesis in the brain by the combined action of VEGFR-1 and VEGFR-2, where the activation of the latter increases angiogenesis, and the activation of the former decreases it (for details see below). Together these receptors ensure that sprouting angiogenesis in the brain is a carefully regulated process. When VEGF-A binds to VEGFR-2, phosphoinositide 3-kinase (PI3K) is activated; this kinase is a central component in the angiogenic process. The molecular link between VEGFR-2 and PI3K is not well described, but Axl, a member of the TAM family of receptor tyrosine kinases, seems to be involved [51]. PI3K activates Kinase B (Akt) [52], which promotes migration of the endothelial cells of the BBB [53,54,55,56]. Activation of the Akt pathway by VEGF-A after stroke has been extensively demonstrated [57,58,59,60]. In a recent study [61], CRISPR/Cas9-mediated depletion of VEGFR-2 was shown to completely block VEGF-induced phosphorylation of Akt in human retinal microvascular endothelial cells. Consequently, the proliferation, migration and tube formation of these cells in vitro was inhibited. This demonstrates the dependency of angiogenesis on the VEGFR-2-PI3K-Akt pathway.

**Figure 1:**
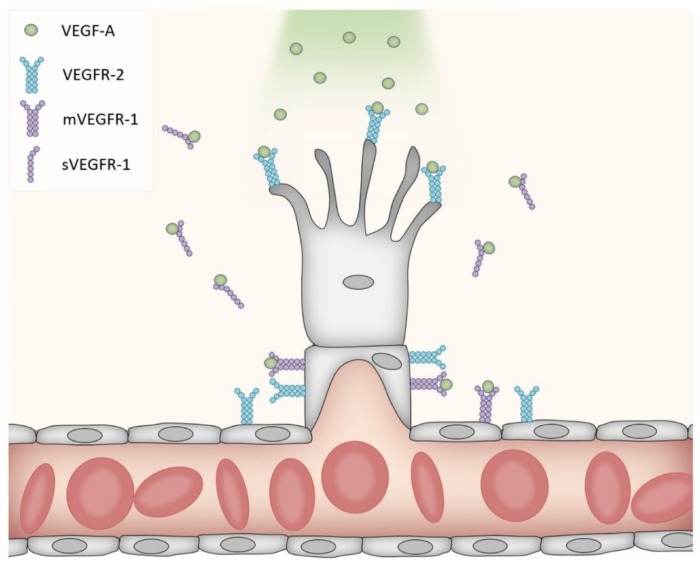
VEGF-A-mediated vascular sprouting. VEGFR-2 is expressed on endothelial cells (shown in gray) including proliferating cells, where it binds VEGF-A, which then induces sprouting. One form of VEGFR-1 is expressed on mature endothelial cells, while another form is secreted (sVEGFR-1). Both forms of VEGFR-1 bind VEGF-A, hence preventing the binding of VEGF-A to VEGFR-2 on non-sprouting parts of the blood vessel (decoy function). This is important to guide the growing vessel in the right direction and prevent the sprouting of neighboring cells.

Further downstream mechanisms of phosphorylated Akt (pAkt) include the activation of nitric oxide synthase (NOS). This enzyme catalyzes the conversion of the amino acid l-arginine to nitric oxide (NO). Four isoforms of NOS are described: endothelial NOS (eNOS), inducible NOS (iNOS), neuronal NOS (nNOS) and mitochondrial NOS (mtNOS) [62,63]. pAkt-induced phosphorylation of eNOS at Ser^1177^, with the subsequent increase in NOS activity, may regulate cerebrovascular functions through several mechanism (for a review of eNOS in cerebrovascular diseases, see [64]). While the role of VEGFR-2 in angiogenesis is well described, the detailed mechanism involved in VEGFR-1 signaling is less known. A reduction of VEGFR-2-mediated pathways seems to be an important effect; alternative splicing of VEGFR-1 results in a membrane-bound form and a soluble form. The latter is secreted from endothelial cells and may modulate the amount of VEGF-A available for binding to VEGFR-2 [65,66]. In addition, VEGFR-1 in the membrane of endothelial cells antagonizes the angiogenic function of VEGFR-2 on the same cells, and VEGFR-1 activation thereby limits vascular growth [67,68]. VEGFR-1 on endothelial cells binds VEGF-A with high affinity, but displays low kinase activity. In fact, deleting the kinase domain without affecting the ligand binding region produced no detectable abnormalities in the density of blood vessels [69]. However, the genetic deletion of VEGFR-1 led to vessel overgrowth and the formation of dysfunctional vessels [68,70]. VEGFR-1 knock-out mice died early in the embryonal period [69], highlighting the importance of VEGFR-1 in addition to VEGFR-2 for proper vascularization. While the secreted VEGFR-1 isoform, but not the membrane-bound isoform, regulates branching [71], both isoforms regulate the mitotic properties of endothelial cells. The current belief is therefore that the secreted VEGFR-1 inactivates VEGF-A on both sides of the sprout, thereby providing the path of higher VEGF-A concentration that guides the sprouting vessel in the proper direction (Figure 1).

Interestingly, VEGF-mediated angiogenesis seems not to be restricted to the ischemia area, as an increase in VEGF-A and a corresponding vascularization have been observed even in the contralesional hemisphere. In fact, Wang et al. [72] found that VEGF-A-induced angiogenesis may lead to a hemodynamic steal phenomena where blood flow is reduced in the ischemic areas but increased in areas outside the lesion [72]. They suggest that VEGF-A protects neurons from ischemic cell death by a direct action on the neurons rather than only by promoting angiogenesis.

In addition to the endothelial cells, other cell types also express VEGF-A receptors, and may contribute to angiogenesis. Pericytes, the contractile cells that wrap around the abluminal surface of the endothelial cells of the vessels, also express VEGFRs. These cells are suggested to play a role in the formation of stable vascular networks. The inhibition of pericyte-specific VEGFR-1 signaling results in the loss of branches and the enlargement of vessels, suggesting that pericytes promote endothelial sprouting [73]. This has only been reported in the retina; the extent to which the pericytes regulate angiogenesis in the brain in response to stroke remains to be investigated.

#### 3.2.2. Effects of VEGF-A on Vasodilation

Outside the central nervous system (CNS), VEGF-A has been shown to have a vasodilative effect, increasing blood flow when expressed under ischemic conditions. For instance, in an ischemic limb model in rabbits, it was shown that the co-application of VEGF-A with serotonin in the iliac artery increased blood flow by more than 100% [74]. In isolated coronary arteries, VEGF-A leads to a slow rise of cytosolic calcium in endothelial cells and an endothelium-dependent relaxation of the arteries [75]. As described above, VEGF may activate the VEGFR-2–PI3K–Akt–eNOS pathway to induce angiogenesis. However, the same pathway is also involved in more acute effects. For instance, eNOS is responsible for vasodilation after hypoxia/ischemia, leading to increased cerebral blood flow [76]. This effect is believed to be mediated through cyclic guanosine monophosphate (cGMP), which escapes from the endothelial cells and causes the relaxation of smooth muscle cells in the vicinity [77]. Furthermore, a systematic review of the effects of NO donors in animals models of stroke [78] concluded that NO donors improved cerebral blood flow and decreased the infarction volume. Further demonstrating the relationship between eNOS and stroke progression, eNOS knock-out mice displayed decreased cerebral blood flow and developed larger cerebral infarctions than wild-type mice [79]. Furthermore, during the first 30 min after a middle cerebral artery occlusion (MCAO) in rats, the administration of the NO precursor l-arginine, or NO donors (sodium nitroprusside (SNP) and 3-morpholino sydnonimine) improved cerebral blood flow and prevented tissue necrosis [79,80,81]. Even though high levels of VEGFR-2 and eNOS were reported 1–3 days after MCAO [82], an acute increase in blood flow in the brain in response to VEGF-A was not detected [83,84]. One explanation may be that a possible vasodilative effect of eNOS is counteracted by capillary pericyte constriction in ischemia [85].

#### 3.2.3. Acute Effects of VEGF-A on Vasopermeability

**Figure 2:**
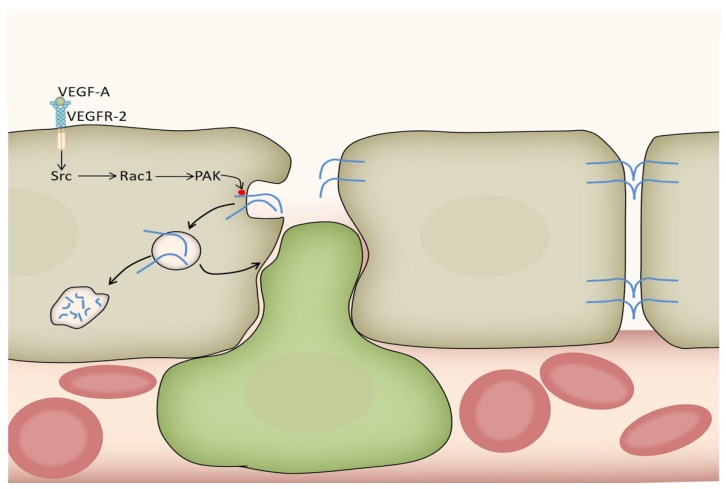
VEGF-A mediated disruption of the blood brain barrier (BBB). VEGF-A binds to VEGFR-2 on endothelial cells (shown in gray) of the BBB leading to the activation of the VEGFR-2–Src–Rac1–PAK pathway. Activated PAK phosphorylates (red dot) the internal tail of the cell-adhesion molecule VE-cadherin (blue), leading to its internalization and subsequently to the disruption of the intercellular junctions of the BBB. Internalized VE-cadherin is then either recycled to the membrane or degraded. The green cell exemplifies a systemic immune cell that is allowed to enter the brain through the fenestrated vessel wall along with other molecules that are normally prevented from entering when the BBB is intact. An intact adherent junction between two endothelial cells in the absence of a VEGF-A signal is shown to the left.

Increased vascular permeability is an early event in stroke. Leaky blood vessels are known to induce edema, which in turn hampers perfusion and therefore result in more pronounced neuronal death [86]. This effect is largely mediated through the action of VEGF-A–VEGFR-2 and the Src pathway, although activation of the PI3K–Akt–eNOS pathway also plays a role in the increased permeability of the BBB seen in acute stroke [87]. The non-receptor tyrosine kinase Src is transiently upregulated in the ischemic brain as early as 3 h after reperfusion [33]. The family of Src kinases consists of proto-oncogenic, non-receptor tyrosine kinases. Src-activation is regulated by a number of different signals, including VEGF-A receptor actions. An increase in Src phosphorylation during the acute phase of ischemia is associated with VEGF-induced vascular permeability [33,88,89]. Src activation then returns to basal levels within the first day, before a second increase occurs 3–7 days after reperfusion [33]. The association of the Src pathway with VEGF-A seems to be bidirectional: under ischemic conditions; Src can regulate the expression of VEGF-A [90], as inhibition of Src decreases VEGF-A levels [33] and consequently VEGF-A-induced vascular permeability [91]. The result is reduced brain edema and reduced lesion volume [92,93]. On the other hand, Src knock-out mice as well as wild-type mice treated with an Src inhibitor are resistant to VEGF-induced vasopermeability and edema [88,91,94]. The latter findings indicate that Src acts downstream of VEGF-A as opposed to the other way around. Activation of the VEGF-A–Src pathway may underlie the unfavorable effects of VEGF-A in the early treatment of stroke. One important VEGF-A–VEGRF-2–Src-mediated mechanism that underlies vasopermeability is the regulation of the adhesion junctions and tight junctions between endothelial cells (Figure 2). For instance, VEGF-A triggers endocytosis of a key cell-adhesion molecule, VE-cadherin, via a VEGFR-2–Src pathway that involves the subsequent phosphorylation of the small GTP-binding protein Rac and the GTPase-activated kinase PAK (p21 activated kinase). Activated PAK phosphorylates the internal tail of VE-cadherin, leading to its internalization and subsequently the disruption of the intercellular junctions of the BBB [95,96,97,98]. In addition to endothelial cell-derived VEGF-A, astrocyte-derived VEGF-A may also contribute to BBB leakage in early stroke, as it has been demonstrated in cultures that ischemic neurons activate astrocytes to increase their VEGF-A production, which in turn induces endothelial barrier disruption [99].

In the context of VEGF-A-mediated BBB disruption early in stroke, inflammation may be an important factor. The neuroinflammatory response after stroke contributes to neural damage, but also plays an important role in neurogenesis, as reviewed in [100]. As mentioned above, VEGF-A seems to be upregulated in response to inflammatory cytokines in the CNS [42,43]. Growing evidence suggests that the ERK pathway contributes to neuroinflammation and neuronal death in ischemic stroke [101], possibly via the regulation of pro-inflammatory cytokines [102]. More research is needed to unravel the direct involvement of VEGF-A in neuroinflammation after stroke.

#### 3.2.4. Effects of VEGF-A on Neuroprotection

Despite the name, VEGF-A does not only act on the vascular endothelium. Instead, VEGF-A acts on several other cell types, including neurons [103]. This has been demonstrated in numerous studies using a diverse array of neuronal preparations [30,104,105,106,107,108]. VEGF-A promotes neuronal survival in cell culture models of stroke, including the oxygen-and-glucose deprivation model [105] and the excitotoxicity model [109,110]. Most of these direct neuronal effects of VEGF-A have been ascribed to the activation of the PI3K-Akt pathway described above, and the mitogen-activated protein kinase (MAPK) cascade. The latter involves the MAP kinase kinase (MEK) and its effector MAP kinase/extracellular signal-regulated kinase (ERK). Both pathways are activated by several signals, including in response to VEGF-A-induced activation of VEGFR-2 [58]. The effect of the MEK–ERK pathway on neuroprotection remains controversial, as the stimulation of cell growth and proliferation [111] as well as neuronal death [112,113,114,115] by this pathway have been described. In vivo, neuroprotective effects of VEGF-A have also been demonstrated in MCAO models of stroke. Local application of VEGF-A to the surface of the reperfused brain reduced the infarction volume in rats [116]. Further demonstrating a protective effect of VEGF-A, the intraventricular infusion of an anti-VEGF-A antibody led to an increased lesion volume [117]. From the in vivo studies, it is not possible to distinguish the direct protective actions of VEGF-A acting on neuronal VEGFRs from the indirect effects mediated through the endothelial VEGFRs.

#### 3.2.5. Neurogenesis

Neurogenesis in the adult brain occurs in two niches: the subventricular zone (SVZ) of the lateral ventricles and the subgranular zone (SGZ) of the dentate gyrus. Although a recent study [118] challenged the concept of adult neurogenesis in the SGZ of humans, most studies show that both niches are sources of neurogenesis throughout adulthood [119,120,121,122,123,124]. Cerebral ischemia stimulates neurogenesis in both of these niches [125,126]. An increased VEGF-A level is probably an important elicitor, as enhanced VEGF-A alone induces neurogenesis in both of these regions [127,128,129]. In transgenic mice that overexpress VEGF-A, not only neurogenesis, but also the migration of newly formed neurons to the peri-infarcted cortex, is increased [128]. This suggests that VEGF-A-induced neurogenesis can replace some of the neurons that die during a stroke. Many reports of neurogenesis describe increased levels of the neural proliferation marker BrdU and the immature neuronal marker doublecortin in the dentate gyrus of the hippocampus as a result of increased VEGF [130,131,132]. Hippocampal neural stem and progenitor cells (NSPS) even may produce VEGF-A in order to maintain the NSPC pool in the subgranular zone [133]. The proliferative actions of VEGF-A seem to require the activation of both ERK and Akt signaling cascades [132].

VEGFR-2 is the main VEGF-A receptor involved in neurogenesis [105,130,134,135]. After cerebral ischemia, neuroblasts expressing VEGFR-2 migrate along vessels in the ischemic area. Furthermore, the blockage of VEGFR-2 reduced neurogenesis in an animal model of stroke [134]. VEGF-A stimulated the expansion of neural stem cells, whereas the blockage of VEGFR-2 activity reduced neural stem cell expansion [135]. Increased numbers of migrating and developing neurons in the penumbra correlated with VEGF-A and VEGFR-2 [49]. VEGF-A is colocalised with the DNA repair factor ERCC6 in neurons but not in astrocytes after MCAO [136], suggesting a direct role in neuronal repair. The inhibition of astrocytes with fluorocitrate reduces VEGF-A-mediated increases in neuronal proliferation markers in newborn neurons after MCAO, suggesting that the VEGF-mediated increase of newly generated neurons is caused by the transdifferentiation of astrocytes into neurons [137].

### 3.3. VEGF-A in Stroke Prevention—Exercise and Preconditioning

#### 3.3.1. Timing and Dosage

**Figure 3:**
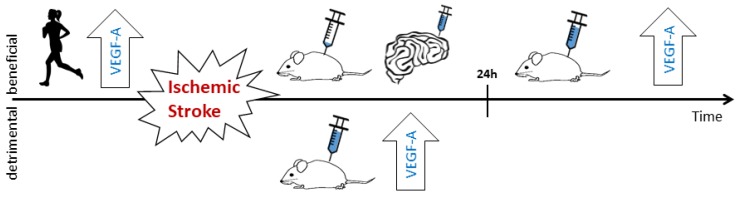
Effects of VEGF-A in cerebral stroke–timeline. Before stroke onset, the upregulation of VEGF-A (large arrow labelled VEGF-A), preconditioning or exercise decreases the risk of stroke as well as the outcome after stroke. The latter is, at least partly, due to the increased formation of collateral (angiogenic effects) and direct neuroprotective effects of VEGF-A. In the acute phase (0–24 h after stroke onset), the systemic administration of VEGF-A at levels leading to angiogenesis, or the intrinsic upregulation of VEGF-A lead to a leaky BBB and corresponding detrimental effects. The application of low non-angiogenic doses (e.g., via a cerebral artery) as well as the intraventricular or topical application of VEGF-A have a neuroprotective effect, even in the acute phase. In the later phase (>24 h) after stroke, increased levels of VEGF-A decrease stroke-induced neural damage.

As described, VEGF-A induces both detrimental (BBB disruption) and beneficial (angiogenesis, neuroprotection and neurogenesis) processes in the ischemic brain. Therefore, whether VEGF-A is neuroprotective or neurotoxic depends on which of these processes dominate. The timing, the dosage and even on the route of administration of VEGF-A after stroke have an influence on the outcome (Figure 3).

Excessive levels of VEGF-A early after stroke increase BBB leakage in the ischemic brain, causing edema and subsequently elevated intracranial pressure that obstruct blood supply (as presented above). In addition, leaky vessels in the penumbra disturb the homeostasis of the nervous tissue, as the molecules and immune cells that are normally prohibited from entering the brain may now pass the BBB. Together, these mechanisms may aggravate neural damage [11,57]. To avoid detrimental effects, intravenous VEGF-A should not be administered between 1–3 and 24 h after stroke onset [11,57]. VEGF-A application later than one day after stroke onset seems to always lead to neuroprotection, increased vascular volume, decreased lesion volume, enhanced neural cell proliferation; even behavioral recovery from stroke is improved [8,127,138].

The route of VEGF-A administration seems to make a difference, as topical (directly on the cortical stroke area) or intracerebroventricular application prevents neural damage as well as BBB leakage, even when applied early after stroke [57,116]. Systemic administration of VEGF-A, as described above, often causes more of the unwanted effects. Furthermore, it has been shown that low doses (less than 2.4 ng/day) infused into the internal carotid artery do not affect endothelial proliferation or changes in vascularization [135,139] and that high doses (~10 ng/day) may lead to neural damage, despite eliciting increased vascularization [139].

#### 3.3.2. Hypoxic/Ischemic Preconditioning

The central principle in preconditioning is that mild forms of stress induce tolerance to an otherwise lethal injury (reviewed in [140]). Hypoxic/ischemic preconditioning means that a brief episode or a mild form of hypoxia/ischemia prior to a stroke will reduce the damage produced by the stroke, and was first described by Kitagawa and co-workers [141]. In laboratory animals, this type of preconditioning is well known to increase the resistance of the brain to hypoxic/ischemic insult [142,143,144,145,146,147]. However, the translation of the preconditioning research to the human setting is challenging. First of all, the average stroke patient is elderly, often suffers from other diseases and uses medications, all of which are factors that may influence the efficacy of the preconditioning. Secondly, the fact that a stroke may occur without warning makes it difficult to administer the preconditioning at a suitable time prior to the stroke. For these reasons, preconditioning in the prevention of stroke has a limited place in clinical practice and the optimal preconditioning strategy remains to be established.

The underlying mechanisms of hypoxia/ischemia-induced preconditioning involve an increase in HIF-1α [148] and its target genes *EPO* and *VEGF*, leading to vascularization [36,149]. Hypoxic preconditioning elicits HIF-1α-dependent upregulation of genes, including VEGF-A, not only during the preconditioning period, but also at an elevated rate during a subsequent ischemia, suggesting that the treatment modifies the brain’s genomic response to ischemia [150]. Ischemic preconditioning in vivo has been shown to protect the hippocampus from ischemic/reperfusion damage by increasing both the expression of VEGF-A and VEGFR-2 [151]. Hypoxic preconditioning in vitro leads to increased levels of VEGF-A, VEGFR-2, pAkt, and pERK in neurons, and the inhibition of VEGFR-2 negates the activation of Akt [152]. Elevated levels of VEGF-A are associated with an increase in collateral formation [153,154], reducing the extent of perfusion-loss in stroke. In line with this, preconditioning by VEGF-A injections increases cerebral perfusion, reduces stroke-induced neural damage [155], and increases neurogenesis even for months after the treatment [156]. Furthermore, the preconditioning event does not need to be present in the organ that is protected, as remote ischemic preconditioning (rIPC) also protects organs from ischemic damage by raising systemic VEGF-A levels [157]. Laboratory experiments have shown that rIPC reduces brain infarction [148,158,159,160]. Two pilot clinical trials have confirmed that rIPC is feasible in people at risk of stroke, and significantly reduces stroke prevalence [161,162].

The fact that a mild or short ischemic/hypoxic event can initiate protective mechanisms that prepare the brain for a larger event of the same kind is comprehensible. However, it appears that most events that produce mild stress in the brain induce ischemic tolerance, regardless of the type of stress [163,164,165,166]. Volatile anaesthetics, for example, induce ischemic tolerance without causing hypoxia/ischemia (reviewed in [167]). The mechanism behind the protective effects of volatile anaesthetics is largely unknown, but HIF-1α seems to be a key mediator also in this context. The involvement of VEGF-A during preconditioning with volatile anaesthetics is also unknown. One study suggested that an increase of VEGF in the acute phase of ischemia after such preconditioning may underlie part of the protective effect [168].

In summary, the HIF-1α–VEGF-A–VEGFR-2–Akt pathway is part of the protective mechanism in hypoxic preconditioning, but one has to keep in mind that a number of additional factors including a number of additional HIF target genes and heat-shock proteins are also involved [169].

#### 3.3.3. Exercise

Exercise is one of the best preventive strategies in stroke, as it induces some of the same mechanisms as seen in hypoxic/ischemic preconditioning. Therefore, exercise may be seen as a means of preconditioning in itself. Pre-ischemic exercise leads to increased VEGF-mediated angiogenesis and reduced brain damage after ischemic stroke [170,171,172,173,174]. The underlying mechanisms are not completely understood, but an increase in eNOS seems to be important [175,176,177,178]. Exercise and oxygen-glucose-deprivation (OGD) induce VEGF-A/VEGFR-2-mediated cAMP response element-binding protein (CREB) phosphorylation as a shared pathway in the protection of both endothelial cells and neurons [179]. In animal studies, treadmill exercise has been reported to be more efficient than exercise in running wheels at inducing protection against stroke [180], suggesting that higher intensities are needed. Lactate, a partial exercise mimetic [181] may be involved. We have recently shown that the lactate receptor HCAR-1 in the brain [182] is responsible for the increased VEGF-A levels and angiogenesis induced by exercise or lactate injections [44].

## 4. VEGF-A in Human Cerebral Stroke

Despite a number of publications discussing the clinical use of VEGF in cerebral stroke, the data supporting a protective role of VEGF-A is almost exclusively based on animal models. Clinical studies are sparse and mainly focus on intrinsic VEGF levels as biomarkers for progression after cerebral stroke. Serum VEGF-A levels in humans increase after stroke [183,184], however, how VEGF-A levels correlate to the severity of the stroke remains to be elucidated. One study found that increased VEGF-A levels might be used as a predictor for improved stoke recovery [185]. Another study, however, found that VEGF-A levels correlated positively with stroke severity in cardioembolic infarction, while a negative correlation to neurological severity was found in atherothrombotic infarction [184]. The current data therefore makes it difficult to determine in which settings VEGF-A manipulation would be beneficial. Additional clinical studies have been performed (e.g., ClinicalTrials.gov identifier: NCT02157896 and NCT00134433), but the results have not been published yet. The challenge in using VEGF-A manipulation in clinical trials probably lies in the multifaceted action of VEGF-A described above. Before VEGF-A can be safely manipulated in a clinical setting, the time window in which the beneficial effects of increased VEGF-A outweigh the detrimental effects needs to be identified. The same is true for the optimal dosage and the route of administration.

## 5. Conclusions

Stroke results from the occlusion of a precerebral or cerebral artery or an intracerebral hemorrhage, both of which lead to focal hypoxia/ischemia and necrosis of the ischemic strike core. Cells in the penumbra may be rescued if adequate perfusion is restored in time. Growth factors affect the recovery after stroke. VEGF-A is a key regulator of angiogenesis, neuroprotection, and neurogenesis. In animal models of stroke, treatment with VEGF-A per se, or with medications that augment VEGF-A effects, reduces the lesion volume. However, VEGF-A treatment has shown somewhat inconsistent results. The timing of the VEGF-A increase as well as the route of administration are important factors to consider when judging the effectiveness of VEGF-A treatment in stroke. During the acute phase, increased VEGF-A induces BBB breakdown and vascular leakage, which lead to disturbed homeostasis, the invasion of peripheral immune cells, and edema. These harmful effects of VEGF-A on vessel integrity are transient, as both VEGF-A preconditioning and increased VEGF-A after the acute phase has a neuroprotective effect (Figure 3). VEGF-A therefore has a Janus face in the treatment of stroke. Further investigations are needed to increase the safety of VEGF-A treatment and to find strategies to enhance the angiogenic, neuroprotective and neurogenic properties, while avoiding the detrimental effects.

## Abbreviations

VEGF	Vascular Endothelial Growth Factor
ERK	Extracellular Signal-Regulated Kinase
PI3K	Phosphoinositide 3-Kinase
HIF	Hypoxia Inducible Factor
